# Unmasking the truth: Experimental evidence of facemask compliance in Bangladesh, Kenya, and Nigeria during the COVID-19 pandemic

**DOI:** 10.1371/journal.pgph.0001086

**Published:** 2023-03-30

**Authors:** Karen A. Grépin, Valerie Mueller, Nicole Wu, Atonu Rabbani

**Affiliations:** 1 School of Public Health, University of Hong Kong, Hong Kong SAR, China; 2 School of Politics and Global Studies, Arizona State University, Tempe, Arizona, United States of America; 3 Department of Economics, University of Dhaka, Dhaka, Bangladesh; 4 James P. Grant School of Public Health, BRAC University, Dhaka, Bangladesh; Pontificia Universidad Católica de Chile: Pontificia Universidad Catolica de Chile, CHILE

## Abstract

High levels of compliance with public health measures are critical to ensure a successful response to the COVID-19 pandemic and other public health emergencies. However, most data on compliance are self-reported and the tendency to overreport due to social desirability could yield biased estimates of actual compliance. A list experiment is a widely used method to estimate social desirability bias in self-reported estimates of sensitive behaviours. We estimate rates of compliance with facemask mandates in Kenya, Nigeria, and Bangladesh using data from phone surveys conducted in March-April 2021. Data on compliance were collected from two different survey modules: a self-reported compliance module (stated) and a list experiment (elicited). We find large gaps between stated and elicited rates of facemask wearing for different groups depending on specific country contexts and high levels of overreporting of facemask compliance in self-reported surveys: there was an almost 40 percentage point gap in Kenya, 30 percentage points in Nigeria, and 20 percentage points in Bangladesh. We also observe differences in rates of self-reported facemask wearing among key groups but not using the elicited responses from the list experiment, which suggest that social desirability bias may vary by demographics. Data collected from self-reported surveys may not be reliable to monitor ongoing compliance with public health measures. Moreover, elicited compliance rates indicate levels of mask wearing are likely much lower than those estimated using self-reported data.

## 1 Introduction

High levels of compliance with public health measures are critical for a successful response to COVID-19 [1]. At the beginning of the pandemic, most countries adopted policies to mandate non-pharmaceutical interventions (i.e. facemasks and social distancing) to mitigate the spread and impact of the virus, many of which require substantial behavior change–an important public health challenge. Adding to the challenge, the prolonged nature of the pandemic meant that it was also important to maintain high levels of compliance with these measures over extended periods of time.

Facemasks are effective, low-cost measures that can be adopted to reduce the transmission of COVID-19. In June 2020, the World Health Organization (WHO) issued guidance to recommend their widespread use to prevent transmission [2], despite the lack of rigorous evidence of their effectiveness among the public at the time. Since then, a meta-analysis of available studies suggest that facemasks are effective at reducing the odds healthcare workers became infected with COVID-19 [3]. A randomized trial conducted in Bangladesh has confirmed that facemasks can reduce the number of symptomatic COVID-19 cases at the population [4]. More recently, a systematic review of randomized trials also found that facemasks were effective at reducing the incidence of all forms of respiratory illness in community settings, and among adults, although the unadjusted results across all the studies identified in the review were not statistically significant [5]. As facemask wearing is relatively harmless, some have also argued that it should be adopted based on the precautionary principle [6]. In low- and middle-income countries (LMICs), the mandated use of facemasks has also been argued as an alternative to much costlier control strategies [7]. Due to these factors, as well as growing evidence supporting their effectiveness and the apparent success of jurisdictions with universal masking mandates, many countries adopted facemask mandates or recommended their use during the first few months of the pandemic, including in LMICs [8–12].

To be effective, however, facemasks need to be worn and worn properly. While studies generally find high levels of compliance with facemask mandates, given that it is socially desirable for people to wear a facemask, it is not clear if self-reported data are reliable. This may be especially true in LMICs where resources to enforce mask mandates may be more limited. While studies in many LMICs have generally found high rates of self-reported compliance [7], one study in Kenya found that self-reported compliance estimates were much higher than estimates made from direct observation in public spaces over the same period [13]. Achieving high rates of compliance is critical to reducing transmission, however, accurate measurement of actual compliance with public health directives is also essential to monitor policy effectiveness. In Kenya, Nigeria, and Bangladesh, the setting for this experiment, surveys conducted during the pandemic have generally found facemask compliance to be 80%-90% and public awareness of the importance of wearing a facemask to be nearly universal [13–16].

List experiments, also known as item count surveys, have frequently been used to overcome social desirability reporting bias in surveys [17]. In such experiments, respondents are shown a list of behaviors with a potentially sensitive behavior randomly included or excluded and are then asked to report how many of the behaviors, but not which ones, they have engaged in over a fixed recall period. As it is not possible to directly infer which of the behaviors the respondent has engaged in, it is believed that respondents have less incentive to lie about sensitive behaviors. The difference-in-means estimator can then be used to generate unbiased estimates of the rates of the sensitive behaviors for the sample. List experiments have been used to measure intimate partner violence, condom use, and abortions [18–21]. They have also been used to study compliance with some public health measures in high-income countries during the pandemic, for example, to measure social distancing in a set of high-income countries and handwashing in Ireland [22, 23]. But to our knowledge, they have not been used to estimate facemask wearing compliance during the pandemic nor in a LMIC setting.

In this paper, we use a list experiment to estimate actual compliance with facemask mandates among people living in three LMICs during the COVID-19 pandemic. At the time of our survey, all three countries had mandatory facemask policies in place: in Kenya, the Ministry of Health mandated masks in public places starting in early April 2020 [24], the Nigerian President mandated everyone to wear a mask in public starting in late April 2020, although similar policies had already been adopted in most states before this policy change [25], and in late May a similar policy had been put into place in Bangladesh [5].

## 2 Materials and methods

### 2.1 Data

Data for this paper come from longitudinal phone surveys that were conducted in Bangladesh, Kenya, and Nigeria during the COVID-19 pandemic. The surveys had been designed to study the gendered effects of the pandemic on a broad set of health, economic, and social outcomes and has been more fully described elsewhere [26]. In all three countries, the first round of the survey was conducted between October-December 2020 and the second round was conducted between March-April 2021. As the list experiment was only included in the second round of the survey, outcome data were sourced from the second of the survey while the socioeconomic and demographic data of respondents were sourced from both rounds of the survey.

To construct our samples, in Kenya and Nigeria, we used a random digit dial (RDD) sampling technique. In each country, registered mobile phone numbers were obtained from a third-party vendor, which were then randomly called by the enumerators to recruit participants. Given the aim of the study was to investigate the gendered effects of the pandemic, and our assumption was that women were likely to be disproportionately affected, in each country, we aimed to recruit approximately 2000 respondents in the first round and targeted to oversample women so that at least 60% of the sample to be women. In Kenya, we used a referral experiment to oversample women, while in Nigeria we used quota sampling to increase the geographic, age, and gender representativeness of our samples [27]. More details of our sample are available in the following paper [26] and alongside the publicly available version of the dataset [28]. In Bangladesh, instead of RDD, we drew upon a recently completed household survey to construct the sample [29]. Individuals were randomly selected from the original survey, initially targeting a sample size of 1800. We oversampled women to ensure a gender balance. Innovations for Poverty Action (IPA) conducted the surveys in Kenya and Nigeria, while the BRAC James P Grant School of Public Health implemented the survey in Bangladesh. We used sampling weights to adjust for selection bias inherent in the RDD sampling approach, non-response, and attrition over the survey rounds in all countries (S1 Questionnaire).

In all countries, the inclusion criteria were that respondents, either men or women, must be at least 18 years of age, must be able to complete the survey in one of the commonly spoken languages in each country (2 in Kenya, 5 in Nigeria, and 2 in Bangladesh), must be willing to be contacted again in the future, and must be willing to provide contact information and a first name for future identification. In our first round, we randomly surveyed 1822 individuals in Bangladesh (914 men and 908 women), 2038 individuals in Kenya (742 men and 1296 women), and 1969 individuals in Nigeria (823 men and 1146 women). In our second round, we completed follow-up surveys with 1722 people in Bangladesh (94.51% follow-up rate), 1647 people in Kenya (80.81%), and 1608 people in Nigeria (81.92%). Small incentives of approximately $1 USD were paid to respondents upon successful completion of survey to increase participation and to reduce attrition between survey rounds.

### 2.2 Measuring stated facemask compliance

During the second round of the survey, we also directly asked respondents about compliance with a list of public health measures, including facemask wearing, which we define as their stated compliance. Specifically, we asked respondents to report how often in the past 7 days they wore a mask when in public and were given the choices of (1) all of the time, (2) most of the time, (3) about half of the time, (4) some of the time, (5) none of the time, and (6) not applicable, I have not been out in public in the past 7 days. The recall period was selected to be directly comparable to the list experiment. We dichotomized the responses by defining compliance with facemask mandates if the respondent reported wearing a facemask all the time (= 1) or most of the time (= 2). Responses were defined as not being compliant otherwise. In the S1 Questionnaire, we also examine the results by more conservatively defining stated facemask compliance by coding “yes” only when “all of the time” is reported. This leads to a considerable reduction in gaps between stated and elicited non-compliance with wearing masks (see Fig A in S1 Appendix).

### 2.3 Eliciting facemask compliance using the list experiment

In our experimental design, also in the second round of the survey, male and female respondents were randomly assigned to be in either the treatment or the control arm of the list experiment with equal probability. In each group, respondents were told that they would be provided a list of activities, and they were asked to report on how many of the activities they had done over the past 7 days in aggregates (see Table A in S1 Appendix). The control group received a list of activities that included: (1) called a friend or family member, (2) listened to the radio, (3) drove in a car or motorcycle, and (4) sent someone mobile money. The treatment received the same list, however, the option of “didn’t wear a mask when in public”, was included in the list between options (3) and (4) of the control list.

### 2.4 Estimation strategy

We followed a standard list experiment design in which respondents from each country were randomly assigned to either the control or treatment group. Respondents were read a list of behaviours (see Table A in S1 Appendix for more details) but rather than report on individual behaviours, respondents were asked to report on the total number of behaviours among those presented to them that they did over the past 7 days. The control group received a list of 4 non-sensitive behaviours while the treatment group respondents received a list with the same items but with an additional question about facemask wearing inserted.

We defined *T* = 1, if the respondents belonged to the treatment group, or *T* = 0 otherwise. In addition, we defined *X*_*ij*_ = 1 if the *i*-th respondent engaged in the *j*-th behaviour, or 0, otherwise, however, this is not directly observable in the way that respondents are asked to respond. Instead, we observe the total number of behaviours, *Y*_*ij*_(*T*), reported by each respondent over the past 7 days, which varies based on differences in underlying behaviour and treatment status. In other words, $Y_{i}(T)=\sum_{j=1}^{J(T_{i})}X_{ij}$, where, *J*(0) = 4, and *J*(1) = 5.

Using data from both the treatment and control groups, we can estimate the fraction of the population agreeing to the sensitive item using the following difference-in-means estimator:

$$\hat{\delta }=\frac{1}{N_{1}}\sum_{i=1}^{N_{1}}T_{i}Y_{i}(T_{i})-\frac{1}{N_{0}}\sum_{i=1}^{N_{0}}\left(1-T_{i}\right)Y_{i}(T_{i})$$ (1)

Here, *N*_*T*_ is the size of the control (if *T* = 0) and treatment (if *T* = 1) group. Since the treatment group has less incentive to lie about whether they engaged in the sensitive mask wearing behaviour when asked in aggregate, the estimator above gives us an estimate of the implied rate of mask wearing in the study population.

We can also estimate the $\hat{\delta }$, using the following equation:

$$Y_{i}=\alpha +\delta T_{i}+\varepsilon_{i}$$ (2)

Moreover, we can use multivariable regressions to understand how responses to the sensitive items vary by respondents’ characteristics by interacting the treatment assignment (i.e., *T*_*i*_) with the individual characteristics (e.g., *Z*_*i*_) by estimating the following equation:

$$Y_{i}=Z_{i}\gamma +T_{i}\cdot Z_{i}\delta +\varepsilon_{i}$$ (3)

Here, again, we are primarily interested in the vector *δ*. We can estimate the parameters (*γ*, *δ*) using OLS models.

Our estimators are valid if the list experiments comply with three desirable properties. The first one requires valid randomization or a balance between the treatment and control groups. In Table B in S1 Appendix, we present the results of a balance test to evaluate the effectiveness of the randomization in our sample. In all three countries, we observe a good balance between our treatment and control groups on all variables used in our analysis. Although women were overrepresented in the overall sample, we observe no difference in the proportion who are assigned to either the treatment or control groups. The second one requires no design effects. We conduct tests for the presence of design affects in Table C in S1 Appendix and show that there were no issues for Kenya and Nigeria, but in Bangladesh we detected design effects in one of the 10 cases tested. The third one requires no liars or the respondent not changing their reports in presence of the sensitive items. This also calls for checking floor and ceiling effects [16]. Our estimates are not sensitive to the inclusion of floor and ceiling effects in the estimation procedures.

### 2.5 Ethical clearance

The survey and human subject participation were reviewed locally for ethics clearance as well as by respective Institutional Review Board committees at The University of Hong Kong and Simon Fraser University. It also obtained ethical clearance from National Health Research Ethics Committee of Nigeria (NHREC) in Nigeria (NHREC/01/01/2007), BRAC James P Grant School of Public Health IRB at BRAC University in Bangladesh (IRB-13 October’20–043), and Maseno University Ethics Review Committee in Kenya (MSU/DRPI/MUERC/00906/20). Verbal consent was obtained from all respondents prior to completing the survey.

## 3 Results

### 3.1 Summary statistics

In Table 1, we provide the summary statistics for our experimental sample, weighted using the survey sampling weights. The final samples included 1647 respondents in Kenya, 1608 in Nigeria, and 1722 in Bangladesh. In all three countries, the samples were slightly more female (63% in Kenya, 58% in Nigeria, and 55% in Bangladesh). All data are weighted using the sampling weights, which are described in the S1 Questionnaire. The weighted average age of the respondents was 32 in Kenya, 31 in Nigeria, and 40 in Bangladesh. About half of the respondents were married in Kenya (52%) and Nigeria (49%) but over 80% of respondents were married in Bangladesh (83%). Household size was largest in Bangladesh (5.0), followed by Nigeria (4.1), then Kenya (3.3). These differences were partially driven by the fact that there were more children per household in Bangladesh than elsewhere although most of the households in all three countries had children. The sample was slightly more urban in Nigeria (65%) than in the other countries (50% in Kenya and 46% in Bangladesh). Some of the differences between Bangladesh and the other two countries may partially be explained by the RDD sampling strategy used in Kenya and Nigeria.

**Table 1:** Demographic and socioeconomic characteristics of respondents in the first survey round in all 3 countries^1^.

	Kenya	Nigeria	Bangladesh
	(N = 1647)	(N = 1608)	(N = 1722)
**= 1 if female**	0.63 (0.48)	0.58 (0.49)	0.55 (0.50)
**Mean Age in years (SD) [Median]**	31.75 (10.48) [29]	31.20 (10.01) [29]	39.88 (13.56) [40]
**= 1 if currently married**	0.52 (0.50)	0.49 (0.50)	0.83 (0.37)
**= 1 if employed**	0.73 (0.44)	0.64 (0.48)	0.27 (0.45)
**= 1 if experienced an income shock** ^**2**^	0.33 (0.47)	0.10 (0.30)	0.03 (0.18)
**= 1 if living in urban areas**	0.50 (0.50)	0.65 (0.48)	0.46 (0.50)
**= 1 if respondent has children**	0.72 (0.45)	0.70 (0.46)	0.86 (0.34)
**Total number of children**	0.73 (0.90)	0.83 (1.29)	1.76 (1.28)
**Household size in number**	3.27 (1.70)	4.10 (2.74)	5.03 (2.38)
**= 1 if knows people with COVID-19** ^**3**^	0.30 (0.46)	0.08 (0.27)	0.18 (0.38)
**= 1 if considers vulnerable to COVID-19** ^**4**^	0.55 (0.50)	0.18 (0.39)	0.17 (0.38)
**= 1 if decide her/himself to wear mask** ^**5**^	0.67 (0.47)	0.73 (0.44)	0.70 (0.46)
**= 1 if food insecure over last 7 days**	0.49 (0.50)	0.51 (0.50)	0.14 (0.35)
**Score for forward lookingness** ^**6**^	7.84 (3.27) [9]	8.08 (2.82) [9]	3.23 (3.32) [3]
**Respondent’s education categories Kenya**			
Primary or below	16.7%		
Secondary	38.7%		
Tertiary	44.6%		
**Respondent’s education categories Nigeria**			
Secondary		44.0%	
Higher		56.0%	
**Respondent’s education categories Bangladesh**			
Pre-primary			31.7%
Primary			36.7%
SSC or higher^7^			31.6%

Notes

Standard errors are presented in parentheses. All reported values are proportions unless otherwise stated. Median values for age and forward lookingness are in brackets.

^1^ All statistics presented here are from the first survey round carried out in October-November 2020, weighted to adjust for the representativeness of individuals by gender, age range, and rural/urban location using national representative surveys; and weights are applied using inverse probability weighting.

^2^ An income shock is defined as lived in a household where at least one member experienced either 1) job loss, 2) nonfarm business closure, or 3) disruption of farming, livestock, fishing activities in the past 12 months.

^3^ Self-reported responses to whether they personally know anyone that has, or has had, COVID-19, including self, other family members living in the same household, family members living outside of the household, friends/neighbours in the same community, friends living outside of the same community, people from work, including colleagues, boss, clients, etc., and other.

0 Perceived COVID-19 risk of self or any other household member contracting COVID-19.

0 Defined as being the only person to decide wearing face masks to protect again COVID-19 in the household.

0 The forward lookingness is measured through the question “How willing are you to give up something that is beneficial for you today in order to benefit more from that in the future?”. The self-reported scores are on a scale from 0 to 10, where 0 means “completely unwilling to do so” and a 10 means “very willing to do so”.

^7^ SSC stands for Secondary School Certificate, which means Class 10^th^ in Bangladesh.

More people had known people with COVID-19 in Kenya (30%) relative to the other countries (8% in Nigeria and 18% in Bangladesh) and Kenyan respondents were also more likely to report feeling vulnerable to COVID-19 (55% in Kenya but less than 20% in the other countries). Data on education was captured differently in each of the countries, reflecting differences in the national educational systems. The Bangladesh sample was overall less educated with a higher proportion of the sample completing less than secondary school than in the other two countries. We also qualitatively assessed time preference or forward lookingness using an 11-point Likert scale by answering how willing the respondent was to give up something to get benefits in the future [30]. We find comparable scores from Kenya and Nigeria (about 8 in both) with a significant lower score for Bangladesh (3.2).

**Fig 1:**
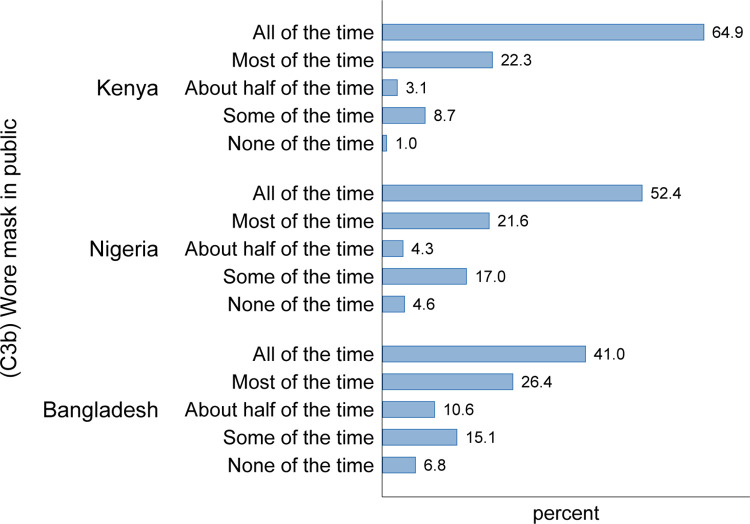
Self-reported mask-wearing in public, by country. Notes: Findings from phones surveys carried out in Kenya (N = 1647), Nigeria (N = 1608), and Bangladesh (N = 1722). The respondents were asked “In the last 7 days, how often did you wear a mask when out in public?”. The tabulations reflect country specific weights.

In Fig 1, we describe the self-reported data on compliance with mask-wearing. In all three countries, there is a high level of self-reported use of mask-wearing and the most common response to this question was to wear a mask “all of the time”. In Kenya, almost 90% of the sample reported using a mask all or most of the time compared to 73% in Nigeria and 66% in Bangladesh. Only 1% of respondents in Kenya, 5% in Nigeria, and 7% in Bangladesh reported wearing a “mask none of the time”.

### 3.2 Comparing stated and elicited facemask compliance

**Fig 2:**
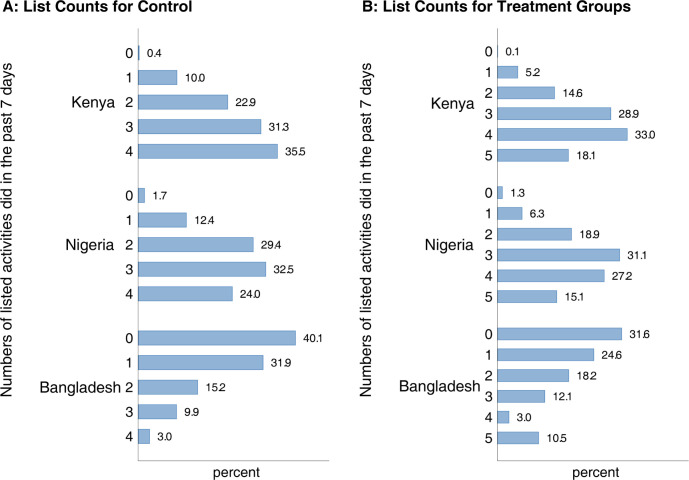
Self-reported number of listed activities, by treatment vs. control groups. Notes: Authors’ calculations from phone survey data from Kenya (N = 1647), Nigeria (N = 1608), and Bangladesh (N = 1722). The questions and designs of the list experiments are described in Table A in S1 Appendix. The tabulations of the number of “yes” answers reflect country specific weights.

In Fig 2, we report the total counts or reported behaviors for the control (panel 2a) and treatment (2b) groups in each country. Although we cannot directly test for the presence of any ceiling or floor effects, we can analyze the distribution of responses to our list experiment questions. In both Kenya and Nigeria, we can see that the distribution to our answers was well distributed with many people answering in the 2–4 answers range and almost no one responded that they did not engage in any of the behaviors. However, in Bangladesh, between 30–40% of the sample in both groups reported having not done any of the behaviors in our sample (see previous discussion of our analysis of the design effects).

**Fig 3:**
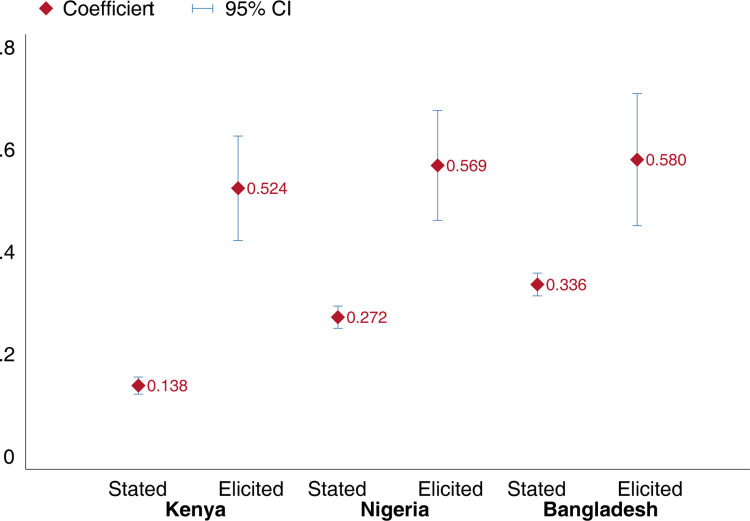
Stated vs. elicited mask wearing rates, by country. Notes: Means and 95% confidence intervals Stated mask non-compliance includes respondents who did not report either “All of the time” or “Most of the time” to the direct question on mask wearing practices over the past seven days. The elicited mask non-compliances are measured using list experiments. All estimates include country specific weights to ensure representability.

In Fig 3, we visually compare stated and elicited estimates of non-compliance with facemask mandates and see a notable difference in all three countries. In Kenya, while only 14% of the sample stated being in public without a facemask over the past week using the data from the self-reported module, 52% of the sample reported not wearing a mask out in public during the past 7 days using the estimates elicited from the list experiment. While not as large as it is in Kenya, important differences were also observed in the other two countries (about 30 and 24 percentage point differences in Nigeria and Bangladesh, respectively).

### 3.3 Multivariable analyses

In Table 2, we report the interaction terms between the coefficients listed and an indicator of whether the respondent was in the treatment group of the list experiment (see Eq 3). In general, based on the practices elicited through the list experiment, we do not find major differences between groups with regards to their levels of compliance in any of the three countries. In Bangladesh, we find those who have autonomy over their mask-wearing, are also less likely to report noncompliance. We also find an education gradient, more educated respondents are more likely to be compliance with facemask mandates in Kenya and Bangladesh, with a statistically significant coefficient in the latter. While there are between-country differences in facemask compliance, the elicited practices generally do not systematically vary within countries.

**Table 2:** Estimates of differences in elicited facemask wearing by socioeconomic and demographic determinants in all 3 countries.

	(1)	(2)	(3)
**VARIABLES**	Kenya	Nigeria	Bangladesh
**= 1 if female**	-0.017	0.133	-0.08
	(0.107)	(0.111)	(0.192)
**Age (in years)**	0.003	-0.007	-0.004
	(0.005)	(0.006)	(0.006)
**= 1 if currently married**	0.115	0.136	0.337
	(0.118)	(0.146)	(0.198)
**= 1 if employed**	0.153	0.007	0.214
	(0.119)	(0.115)	(0.183)
**= 1 if experienced an income shock**	0.008	0.029	-0.05
	(0.108)	(0.174)	(0.290)
**= 1 if living in urban areas**	-0.078	-0.027	-0.015
	(0.103)	(0.112)	(0.149)
**= 1 if respondent has children**	0.066	-0.102	0.359
	(0.129)	(0.136)	(0.236)
**Total number of children**	0.011	-0.072	-0.087
	(0.084)	(0.059)	(0.088)
**Household size**	-0.043	0.032	0.001
	(0.045)	(0.026)	(0.041)
**= 1 if knows people with COVID-19**	-0.007	-0.065	-0.066
	(0.111)	(0.203)	(0.203)
**= 1 if considers vulnerable to COVID-19**	0.006	0.054	0.106
	(0.105)	(0.134)	(0.215)
**= 1 if decide her/himself to wear mask**	0.044	0.056	-0.502**
	(0.109)	(0.127)	(0.185)
**= 1 if food insecure over last 7 days**	-0.029	-0.155	-0.13
	(0.103)	(0.108)	(0.200)
**Score for forward lookingness**	-0.011	0.013	0.028
	(0.016)	(0.019)	(0.023)
**Respondent’s education categories Kenya**			
**Primary**			
**Secondary**	Base		
	-0.241		
**Tertiary**	(0.156)		
	-0.218 (0.162)		
**Respondent’s education categories Nigeria**			
**Secondary**		Base	
**Higher**		0.049	
		(0.110)	
**Respondent’s education categories Bangladesh**			
**Pre-primary**			Base
**Primary**			-0.330
			(0.173)
**SSC or higher**			-0.411*
			(0.174)
**Observations**	1647	1608	1722

Notes: The dependent variable is the score from the list experiment, which was collected during the second survey round. We report here the coefficients of the interaction terms between the baseline characteristics and the treatment indicator. Robust standard errors in parentheses.

*** p<0.01

** p<0.05

* p<0.1. All statistics presented are weighted to adjust for the representation of individuals by gender, age range and rural/urban region using national representative surveys; and weights are applied using inverse probability weighting.

However, there is more systematic within-country variation in stated facemask wearing when respondents are asked to self-report compliance (Table 3). In Kenya and Nigeria, female respondents are more likely to report wearing masks relative to males, so are urban respondents and respondents who knew someone with COVID-19 in Kenya and Bangladesh. In Bangladesh, respondents who are more forward-looking and more educated were also less likely to report not wearing a facemask when in public. The lack of measurable variation in elicited responses via the list experiment but measurable differences in variation in self-reported compliance suggests that social desirability bias is likely to affect some groups more than others.

**Table 3:** Socioeconomic and demographic determinants of stated noncompliance with facemask wearing in all 3 countries.

	(1)	(2)	(3)
VARIABLES	Kenya	Nigeria	Bangladesh
**= 1 if female**	-0.049**	-0.077**	-0.01
	(0.018)	(0.024)	(0.031)
**Age (in years)**	-0.002*	-0.003	-0.002
	(0.001)	(0.001)	(0.001)
**= 1 if currently married**	-0.023	-0.005	0.007
	(0.019)	(0.030)	(0.038)
**= 1 if employed**	0.021	-0.008	0.015
	(0.017)	(0.024)	(0.033)
**= 1 if experienced an income shock**	-0.012	0.024	-0.147**
	(0.017)	(0.039)	(0.052)
**= 1 if living in urban areas**	-0.070***	0.006	-0.130***
	(0.016)	(0.023)	(0.027)
**= 1 if respondent has children**	0.007	-0.003	0.035
	(0.020)	(0.028)	(0.043)
**Total number of children**	0.013	-0.002	-0.011
	(0.012)	(0.011)	(0.017)
**Household size**	-0.003	0	0.001
	(0.006)	(0.005)	(0.007)
**= 1 if knows people with COVID-19**	-0.055**	-0.068	-0.072*
	(0.017)	(0.038)	(0.035)
**= 1 if considers vulnerable to COVID-19**	-0.031	-0.085**	-0.01
	(0.017)	(0.027)	(0.038)
**= 1 if decide her/himself to wear mask**	-0.005	-0.021	0.025
	(0.017)	(0.027)	(0.030)
**= 1 if food insecure over last 7 days**	0.016	-0.01	-0.039
	(0.017)	(0.023)	(0.039)
**Score for forward lookingness**	-0.004	0.003	-0.016***
	(0.002)	(0.004)	(0.004)
**Respondent’s education categories Kenya**			
Primary	Base		
Secondary	0.029		
	(0.026)		
Tertiary	0.033		
	(0.026)		
**Respondent’s education categories Nigeria**			
Secondary		Base	
Higher		-0.019	
		(0.023)	
**Respondent’s education categories Bangladesh**			
Pre-primary			Base
Primary			-0.063*
			(0.031)
SSC or higher			-0.136***
			(0.031)
Observations	1647	1608	1722

Note: The dependent variable is a binary outcome indicating not regularly wearing mask in public, which was collected during the second round of the survey. We are reporting the marginal effects from logit regressions.

*** p<0.01

** p<0.05

* p<0.1. All statistics presented is weighted to adjust for the representation of individuals by gender, age range and rural/urban region using national representative surveys; and weights are applied using inverse probability weighting.

In Table 4, we present the difference of the means of the stated vs elicited estimates of facemask compliance for different sub-groups of the sample. A positive difference is an estimate of the rate of overstatement of facemask wearing in each group. In general, while we find that most groups tend to overreport, there are not many groups that consistently overreport across all the countries. Married people tend to overreport as compared to unmarried respondents and more educated people may be less likely to overreport than less educated people, although the pattern does not strictly hold in Nigeria.

**Table 4:** Comparing stated and elicited facemask compliance by baseline socioeconomic and demographic determinants in all 3 countries.

		Kenya		Nigeria		Bangladesh	
		Difference	p-value	Difference	p-value	Difference	p-value
**Gender**	Male	0.380	0.000	0.170	0.035	0.343	0.004
	Female	0.383	0.000	0.395	0.000	0.233	0.014
**Age** ^a^	Below median	0.256	0.001	0.339	0.000	0.297	0.008
	Above median	0.511	0.000	0.249	0.001	0.186	0.054
**Marital status**	Unmarried	0.267	0.000	0.261	0.001	-0.204	0.198
	Married	0.489	0.000	0.347	0.000	0.340	0.000
**Job status**	Unemployed	0.230	0.024	0.345	0.000	0.224	0.011
	Employed	0.438	0.000	0.253	0.000	0.423	0.004
**Employment shock**	No	0.357	0.000	0.304	0.000	0.263	0.001
	Yes	0.441	0.000	0.275	0.112	-0.079	0.789
**Location**	Rural	0.390	0.000	0.319	0.001	0.220	0.038
	Urban	0.373	0.000	0.294	0.000	0.308	0.007
**Have a child?**	No	0.333	0.001	0.367	0.000	-0.092	0.595
	Yes	0.402	0.000	0.275	0.000	0.316	0.000
**Household Size**	1–2	0.313	0.001	0.221	0.038	-0.208	0.462
	3–5	0.385	0.000	0.301	0.000	0.254	0.001
	6+	0.151	0.463	0.385	0.003	0.285	0.038
**Knows somebody with Covid**	No	0.365	0.000	0.302	0.000	0.277	0.001
	Yes	0.422	0.000	0.297	0.138	0.199	0.311
**Considers him/herself vulnerable to Covid**	No	0.346	0.000	0.272	0.000	0.237	0.004
	Yes	0.436	0.000	0.455	0.000	0.365	0.083
**Decides him/herself whether to wear a mask**	No	0.328	0.000	0.288	0.008	0.604	0.000
	Yes	0.412	0.000	0.310	0.000	0.112	0.189
**Food insecure over past 7 days**	No	0.368	0.000	0.369	0.000	0.288	0.001
	Yes	0.395	0.000	0.212	0.008	0.103	0.629
**Score for forward lookingness** ^a^	Below median	0.407	0.000	0.308	0.000	0.129	0.190
	Above median	0.357	0.000	0.270	0.005	0.330	0.005
**Kenya education categories**	Primary	0.662	0.000				
	Secondary	0.303	0.000	.	.	.	.
	Tertiary	0.356	0.000	.	.	.	.
**Nigeria education categories**	Secondary	.	.	0.259	0.002	.	.
	Higher	.	.	0.321	0.000	.	.
**Bangladesh education categories**	Pre-primary	.	.	.	.	0.312	0.009
	Primary	.	.	.	.	0.280	0.051
	SSC or higher	.	.	.	.	0.173	0.205

Note: In this table, we report the differences in elicited (calculated using the list experiment) and the stated (measured by direct questioning) facemask compliance, stratified by baseline socioeconomic and demographic characteristics.

^a^ Median values are calculated within each country.

## 4 Discussion

High rates of compliance with public health measures are essential to mounting effective responses during infectious disease outbreaks, however, given social pressures associated with the adoption of socially desirable preventive behaviors it is challenging to measure compliance using self-reported data alone. This study demonstrates that there were large and meaningful differences in stated and elicited rates of mask-wearing comparing data from a self-reported module to data from a list experiment in Kenya, Nigeria, and Bangladesh implying social desirability bias likely plagues estimates of this behavior collected in self-reported surveys. Indeed, there was an almost 40 percentage point gap between stated and elicited non-compliance in Kenya. Although smaller in the other countries, the gap was still almost 30 percentage points in Nigeria and 20 percentage points in Bangladesh. In Bangladesh, experimentally induced differences in facemask compliance of approximately 30 percentage points were associated with a reduction in symptomatic seroprevalence of COVID-19 by over 11% [5]. Other studies have also generally found that high levels of compliance are needed to reduce transmission at the community level [4, 31, 32]. Thus, effectiveness facemask mandates to reduce population-level transmission of COVID-19 requires both high levels of mask-wearing–and importantly the ability accurately measure actual compliance. Commonly used self-reported surveys are likely overestimating facemask compliance.

Studies conducted in high-income countries during the pandemic have also shown that estimates of public health compliance may also depend on how survey questions are framed and worded. For example, an online survey conducted in Ireland found lower levels of reported handwashing when questions were worded negatively [23]. A guilt-free survey strategy, in which instead of only asking yes or no questions, respondents were also given the option to report such behaviors ‘occasionally’ or ‘only when necessary’, increased estimates of non-compliance with preventive public health measures by 9–16 percentage points in 12 high-income countries and, separately, in Canada [33, 34]. It is therefore not surprising that we have also identified important survey design effects in our estimates of facemask compliance in lower-income countries. However, the relevance of these findings may be more important from a public policy perspective.

Our study has several limitations which should be taken into consideration when interpreting our findings. First, although our study was conducted similarly in all three countries, there were some important differences in the actual data collection processes across the three countries, including in how the samples were constructed and small differences with regards to the wording of some of the questions across countries. Second, there appear to have been some design effects in the Bangladesh sample due to lower reported rates of the non-controversial behaviors (i.e., floor effects). While it is not possible to determine how much of an effect it may have had in the interpretation of our results or the validity of the study, it does suggest we should interpret the results from Bangladesh more cautiously.

Although the data collected from the compliance and list experiment were not perfectly comparable (one asks about never being out in public without a face mask and the other asked about the frequency of facemask wearing using coarser categories) our findings suggest substantial differences likely do exist between self-reported and actual compliance with facemask wearing in LMIC. Notably, social desirability bias may vary according to gender, location of residence, and education levels. All three countries had facemask mandates in place for many months at the time of our survey, and despite very high rates of compliance reported in self-reported surveys conducted in these countries around the time of our survey, our findings suggest that actual compliance with facemask wearing was far from optimal and likely lower than that estimated in other studies. It is therefore important to understand which factors, above and beyond mandates, are important to help increase actual rates of compliance. This may be especially true in LMIC settings where resources to enforce mandates may be more limited. A recent large scale randomized trial implemented in Bangladesh to test strategies to increase mask usage found that the free provision alone of masks had only a small effect on uptake but that periodic monitoring in public places led to large increases in mask-wearing and that these effects were sustained over long periods [5]. The monitoring intervention also led to increases in other preventive behaviors, namely physical distancing, which further points to the importance of social norms and pro-social learning in the promotion of public health measures. On the other hand, studies have also found that the use of facemasks can reduce compliance with other public health measures, such as social distancing, which could reduce the potential effectiveness of mask wearing [35]. It is therefore also important to consider these social norms in our ability to measure these behaviors and our study demonstrates the need to use measurement strategies to overcome these pro-social biases in surveys. Simple methodological innovations such as a list experiment help to reduce the effect of social desirability in the measurement of such behaviors.

## Supporting information

S1 Appendix Table A: List experiment design. Table B: Balance Test. Table C: Design Test. Fig A: Differences between stated and elicited non-compliance with facemask wearing.
(DOCX)

S1 Data List_exp_data_plos_gh.dta: Processed dataset used in the analysis in STATA format. list01_organizing_variables_v4ar_r2_v1ar.do: STATA do file used to generate processed dataset from raw dataset (not provided but available upon request from corresponding author). list02_symmary_stat_tables_r2_v1ar.do: STATA do file used to generate Table 1: Summary Statistics, Table B: Balance Test, and Table C: Design Test. list03_descriptive_graphs_r2_v1ar.do: STATA do file used to generate Fig 1: Self-reported mask-wearing in public, by country, Fig 2: Self-reported number of listed activities, by treatment vs. control groups, Fig 3: Stated vs. elicited mask wearing rates, by country, and Fig A: Differences between stated and elicited non-compliance with facemask wearing. list04_analyze_list_experiments_r2_v1ar.do: STATA do file used to generate Table 2: Multivariable analyses for the list experiments and Table 3: Multivariable analyses for stated noncompliance with wearing facemask. list05_differences_by_strata_r2_v1ar.do: STATA do file used to generate Table 4: Comparing Stated and Elicited Facemask Compliance.
(ZIP)

S1 Questionnaire Inclusivity questionnaire. (DOCX)
