# Supplementary material for: Unmasking the truth: Experimental evidence of facemask compliance in Bangladesh, Kenya, and Nigeria during the COVID-19 pandemic
Source: PLOS Glob Public Health. 2023 Mar 30;3(3):e0001086. doi: 10.1371/journal.pgph.0001086 (PMC10062667; doi:10.1371/journal.pgph.0001086)
Supplement: S1 Appendix — Table A: List experiment design. Table B: Balance Test. Table C: Design Test. Fig A: Differences between stated and elicited non-compliance with facemask wearing. (DOCX) [file pgph.0001086.s001.docx]

**Unmasking the truth: experimental evidence of facemask compliance in Bangladesh, Kenya, and Nigeria during the COVID-19 pandemic**

**Appendix Paper**

**Survey Weights**

In our analysis, we use sampling weights to adjust for effects of the RDD sampling methodology as well as attrition bias due to the challenges associated with recontacting all baseline respondents. The sampling weights were constructed by Innovations for Poverty Action (IPA) for the Bangladesh, Kenya, and Nigeria surveys using nationally representative surveys. Specifically, we used the 2016 Bangladesh Household, Income, and Expenditure Survey, the 2016 Kenya Integrated Household Budget Survey and the 2019 Kenya Population and Housing Census in Kenya, and the 2018-9 Nigeria General Household Panel Survey in Nigeria. The weights were designed to adjust for the proportion of individuals in our sample relative to what would be expected in a nationally representative survey according to gender, age, region, and phone ownership following the approach adopted by the World Bank [1]. Within-cell post-stratification weights are constructed for the baseline surveys in all countries and then top-coded at the 99th percentile [2]. In Kenya and Nigeria, an attrition correction factor was derived based on the response rate within demographic group bins in the second round [2].

**Table A: List Experiment Design**

| Considering your behaviour over the past 7 days, how many of the following statements are true statements? | | |
| --- | --- | --- |
|  | **Version 1: Control Group** | **Version 2: Treatment Group** |
| Called a friend/family member | 1 = Yes  0 = No | 1 = Yes  0 = No |
| Listened to the radio |  |  |
| Drove in a car or on a motorcycle |  |  |
| Sent someone mobile money |  |  |
| Left the house without a face mask | Not included |  |
| Total possible score | 0-4 | 0-5 |

**Table B: Balance Test**

|  | **Kenya** | | |  | **Nigeria** | | |  | **Bangladesh** | | |
| --- | --- | --- | --- | --- | --- | --- | --- | --- | --- | --- | --- |
|  | **Control** | **Treatment** |  |  | **Control** | **Treatment** |  |  | **Control** | **Treatment** |  |
|  | **(N = 824)** | **(N = 824)** | **p-value** |  | **(N = 765)** | **(N = 848)** | **p-value** |  | **(N = 862)** | **(N = 860)** | **p-value** |
| **= 1 if female** | 0.64 (0.48) | 0.63 (0.48) | 0.784 |  | 0.58 (0.49) | 0.57 (0.49) | 0.894 |  | 0.53 (0.50) | 0.57 (0.50) | 0.093 |
| **Age (in years)** | 31.42 (10.06) | 32.09 (10.88) | 0.194 |  | 31.05 (10.22) | 31.34 (9.82) | 0.557 |  | 39.94 (13.54) | 39.83 (13.58) | 0.859 |
| **= 1 if currently married** | 0.51 (0.50) | 0.53 (0.50) | 0.329 |  | 0.50 (0.50) | 0.48 (0.50) | 0.449 |  | 0.82 (0.38) | 0.84 (0.37) | 0.387 |
| **= 1 if employed** | 0.72 (0.45) | 0.73 (0.44) | 0.711 |  | 0.62 (0.49) | 0.65 (0.48) | 0.163 |  | 0.29 (0.45) | 0.25 (0.43) | 0.064 |
| **= 1 if experienced an income shock** | 0.33 (0.47) | 0.33 (0.47) | 0.869 |  | 0.10 (0.31) | 0.09 (0.29) | 0.422 |  | 0.03 (0.18) | 0.03 (0.17) | 0.546 |
| **= 1 if living in urban areas** | 0.49 (0.50) | 0.52 (0.50) | 0.353 |  | 0.65 (0.48) | 0.64 (0.48) | 0.686 |  | 0.48 (0.50) | 0.44 (0.50) | 0.130 |
| **= 1 if respondent has children** | 0.73 (0.45) | 0.71 (0.45) | 0.492 |  | 0.69 (0.46) | 0.70 (0.46) | 0.420 |  | 0.88 (0.33) | 0.85 (0.36) | 0.073 |
| **Total number of children** | 0.73 (0.89) | 0.73 (0.90) | 0.959 |  | 0.79 (1.34) | 0.85 (1.24) | 0.368 |  | 1.79 (1.25) | 1.73 (1.31) | 0.329 |
| **Household size** | 3.27 (1.74) | 3.28 (1.65) | 0.850 |  | 4.08 (2.80) | 4.12 (2.69) | 0.745 |  | 5.08 (2.46) | 4.99 (2.29) | 0.437 |
| **= 1 if knows people with COVID-19** | 0.30 (0.46) | 0.31 (0.46) | 0.757 |  | 0.08 (0.28) | 0.08 (0.27) | 0.880 |  | 0.19 (0.39) | 0.16 (0.37) | 0.175 |
| **= 1 if considers vulnerable to COVID-19** | 0.57 (0.49) | 0.53 (0.50) | 0.078 |  | 0.19 (0.40) | 0.17 (0.38) | 0.253 |  | 0.18 (0.39) | 0.17 (0.37) | 0.461 |
| **= 1 if decide her/himself to weak mask** | 0.67 (0.47) | 0.67 (0.47) | 0.961 |  | 0.74 (0.44) | 0.73 (0.44) | 0.590 |  | 0.72 (0.45) | 0.68 (0.47) | 0.096 |
| **= 1 if food insecured over last 7 days** | 0.50 (0.50) | 0.49 (0.50) | 0.541 |  | 0.54 (0.50) | 0.49 (0.50) | 0.025 |  | 0.13 (0.33) | 0.15 (0.36) | 0.145 |
| **Score for forward lookingness** | 7.91 (3.27) | 7.76 (3.28) | 0.327 |  | 8.16 (2.80) | 8.01 (2.84) | 0.290 |  | 3.14 (3.37) | 3.33 (3.27) | 0.227 |
| **Respondent's education categories Kenya** |  |  |  |  |  |  |  |  |  |  |  |
| Primary | 16.8% | 16.6% | 0.728 |  |  |  |  |  |  |  |  |
| Secondary | 45.4% | 43.8% |  |  |  |  |  |  |  |  |  |
| Tertiary | 16.8% | 16.6% |  |  |  |  |  |  |  |  |  |
| **Respondent's education categories Nigeria** |  |  |  |  |  |  |  |  |  |  |  |
| Secondary |  |  |  |  | 43.2% | 42.2% | 0.662 |  |  |  |  |
| Higher |  |  |  |  | 56.8% | 57.8% |  |  |  |  |  |
| **Respondent's education categories Bangladesh** |  |  |  |  |  |  |  |  |  |  |  |
| Pre-primary |  |  |  |  |  |  |  |  | 31.1% | 32.3% | 0.849 |
| Primary |  |  |  |  |  |  |  |  | 37.5% | 35.9% |  |
| SSC or higher |  |  |  |  |  |  |  |  | 31.4% | 31.8% |  |

**Table C: Design Test**

|  | Kenya | | Nigeria | | Bangladesh | |
| --- | --- | --- | --- | --- | --- | --- |
|  | $\boldsymbol{\pi}_{\boldsymbol{R}\boldsymbol{,}\boldsymbol{S}}$ | **p-value** | $\boldsymbol{\pi}_{\boldsymbol{R}\boldsymbol{,}\boldsymbol{S}}$ | **p-value** | $\boldsymbol{\pi}_{\boldsymbol{R}\boldsymbol{,}\boldsymbol{S}}$ | **p-value** |
| Pr(R=0,S=1) | 0.002 | 0.842 | 0.004 | 0.746 | 0.092 | 1.000 |
| Pr(R=0,S=0) | 0.001 | 0.842 | 0.013 | 1.000 | 0.320 | 1.000 |
| Pr(R=1,S=1) | 0.050 | 1.000 | 0.063 | 1.000 | 0.160 | 1.000 |
| Pr(R=1,S=0) | 0.050 | 1.000 | 0.058 | 1.000 | 0.152 | 1.000 |
| Pr(R=2,S=1) | 0.133 | 1.000 | 0.167 | 1.000 | 0.121 | 1.000 |
| Pr(R=2,S=0) | 0.096 | 1.000 | 0.126 | 1.000 | 0.026 | 0.891 |
| Pr(R=3,S=1) | 0.157 | 1.000 | 0.183 | 1.000 | 0.102 | 1.000 |
| Pr(R=3,S=0) | 0.155 | 1.000 | 0.143 | 1.000 | -0.001^a^ | 0.464 |
| Pr(R=4,S=1) | 0.182 | 1.000 | 0.152 | 1.000 | 0.105 | 1.000 |
| Pr(R=4,S=0) | 0.174 | 1.000 | 0.091 | 1.000 | -0.077^b^ | 0.000 |

**Fig A: Differences between stated and elicited non-compliance with facemask wearing**


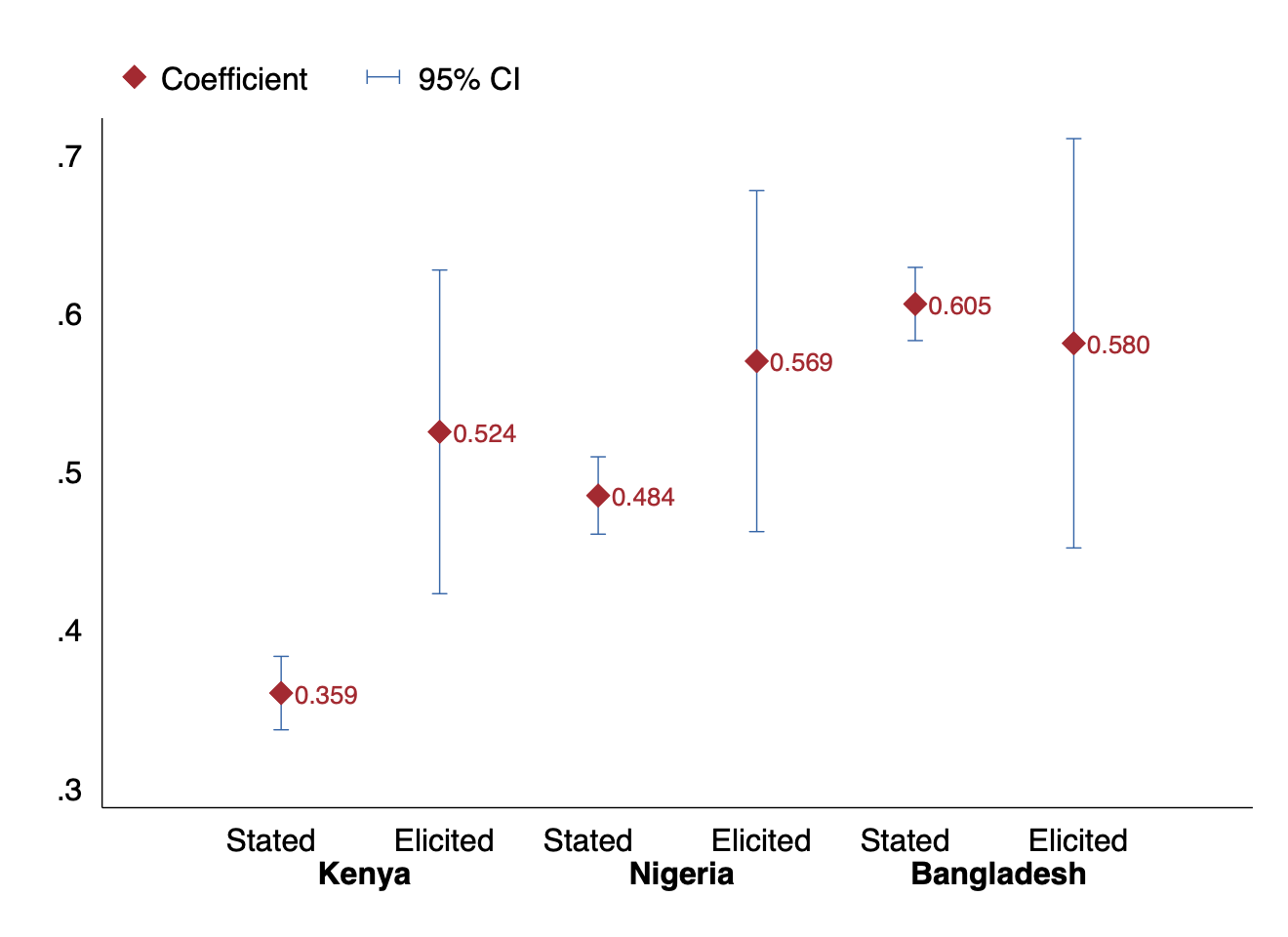


**References**

1. Pape UJ, Sinha N, Delius AJS. COVID-19 Rapid Response Phone Survey Households 2020-2021, Panel [Internet]. Washington, DC: 2020. Available from: https://microdata.worldbank.org/index.php/catalog/3774

2. Kastelic KH, Eckman S, Kastelic JG, Mcgee KR, Wild M, Yoshinda N, et al. High Frequency Mobile Phone Surveys of Households to Assess the Impacts of COVID-19 (Vol. 2) : Guidelines on Sampling Design [Internet]. Washington, DC: 2020. Available from: https://documents.worldbank.org/en/publication/documents-reports/documentdetail/742581588695955271/guidelines-on-sampling-design
